# Determining Positive Behavioral Skills in Different Age Groups of Young Basketball Players during the Pandemic

**DOI:** 10.3390/children10060914

**Published:** 2023-05-23

**Authors:** Eimantas Pocius, Romualdas Malinauskas

**Affiliations:** Department of Physical and Social Education, Lithuanian Sports University, 44221 Kaunas, Lithuania

**Keywords:** young athletes, COVID-19 pandemic, positive personal skills, positive social skills, positive emotional skills

## Abstract

Assessing psychological indicators such as positive behavioral skills in the context of adolescent personality development during the pandemic era is highly relevant: the growing problem of peer disrespect among adolescents who participate in sports has recently become an undeniable scientific issue. This study aimed to analyze positive behavioral skills in the cadet (U16) and junior (U18) age groups of young basketball players during the COVID-19 pandemic. The participants were 378 male athletes (age 16.36 ± 1.15 years). Results revealed that U18 athletes are more capable of taking responsibility, positively evaluating themselves, behaving pro-socially with teammates, cooperating, demonstrating assertiveness, demonstrating self-control, and managing emotions than U16 adolescent athletes. When comparing the effect sizes in the current study during the pandemic with similar studies by other authors, the pandemic may have had a larger negative effect on some positive behavioral skills (ability to control emotions, social responsibility skills, cooperation skills) in U16 athletes than in U18 athletes, as the effect sizes were small before the pandemic and moderate during the pandemic in the current study. This study’s results may be useful for developing and implementing a young athletes’ education program based on a comprehensive model of positive behavioral skills that include the indicators analyzed.

## 1. Introduction

During adolescence, intense and important personal changes take place: an individual is biologically, psychologically, and socially maturing, learning, and becoming capable of living independently [1]. These psychosocial developmental characteristics are determined by the interaction between developmental attainments of previous life stages and biological, social, and cultural factors that emerge during adolescence [2]. Two important psychological processes begin during adolescence: the search for self-knowledge and the pursuit of independence [3]. In adolescence, individuals must become independent adults. This means simultaneously developing a more comprehensive sense of self-identity and establishing stronger relationships with peers at a time of social upheaval. However, during the pandemic, opportunities to strengthen existing or create new social relationships were limited, making it difficult for adolescents to develop the skills necessary for social interaction [4,5].

Essentially, the development of a teenager’s personality begins to stabilize between the ages of 13 and 18, when the behavior of most teenagers becomes more positive [6]. However, adolescence remains characterized by perhaps the most prominent expression of negative behavior compared to other stages of life. Especially around the ages of 15–18, teenagers exhibit behaviors typically related to the search for various sensations and the desire for new and risky experiences [7,8]. Notably, half of all criminals commit their first crime between the ages of 14 and 17 [4]. Between the ages of 13 and 17, teenagers spend increasingly less time with their families and increasing time with peers, meaning that during this period, there is a growing need for teenagers to successfully integrate into an array of relatively unstable social networks without experiencing social exclusion [9].

Around the ages of 13–18, adolescents are typically less satisfied with the attachment possibilities and new social relationships offered by schools, making extracurricular activities such as team sports important [4]. Millions of adolescents around the world engage in sports, and sports are one of the most popular activities for active adolescents in many countries [10,11]. By participating in team sports, adolescent athletes learn to work hard, overcome failures and difficulties, and cooperate with others [12]. When performing sports, adolescents can satisfy their increasing need for social relationships [13]. Adolescents who engage in sports, especially team sports, compete with others to achieve a certain goal. For this reason, they must work together with others, which helps them develop socially [14]. Because team sports are characterized by interaction with other individuals, such games satisfy the growing imperative for social relationships, with the ability to establish one’s athletic competence, allowing for a greater sense of self-esteem [15]. Sports can enhance interpersonal interaction, thereby promoting greater prosocial behavior tendencies [16,17], especially when individuals are highly involved in sports activities for a prolonged period [18,19]. Basketball has been observed to be more beneficial for developing prosocial behavior than other sports [20], with its rules encouraging cooperation [21].

These findings suggest that sports are a fundamental activity for the development of positive behaviors [22,23,24]. However, the competitiveness that is inherent to sports can create social exclusion [25], disrupt close relationships, promote social division, and even encourage social crime among teenagers [8,26,27]. The growing problem of aggression and mutual disrespect among teenagers who engage in sports has been frequently emphasized in the public space and scientific research in recent years, making it an indisputably relevant scientific issue [7,8,28]. It has been suggested that due to the focus on results and consequent competition, teenagers engaged in team sports experience anxiety, which negatively affects their mental health [29,30]. Furthermore, teenagers who engage in team sports also experience emotional violence [31,32,33,34], with research findings indicating that teenage boys engaged in sports exhibit riskier and more socially unacceptable behavior to a greater extent than girls [35,36,37,38,39]. The most pronounced expression of aggression is observed in late adolescence (up until teenagers finish high school) [40,41], and the expression of the typical aggressive and risky behavior characteristic of later adolescence begin to decline around the age of 20 [6].

Positive behavioral skills are defined as an individual’s ability to create personal wellbeing by interacting with other people or groups of people, adapting to the demands of different environments or cultures [42]. Positive behavioral skills include positive personal, emotional, and social skills [43,44]. Positive personal skills are defined as the skills of having a positive relationship with oneself that contribute to one’s personal development; positive emotional skills are understood as the ability to regulate one’s own and other people’s emotions in a way that achieves one’s communication goals and builds and maintains good relationships with others; positive social skills capture the ability to interact in a socially acceptable way [44]. Thus, positive behavioral skills enable a person to maintain positive social relationships, manage their emotions, and manage their behavior [45,46]. Positive behavioral skills are measured using various psychological questionnaires designed to assess skills. For some authors, for practical purposes, skills can also be conceptualized in terms of the measures used to assess them, further emphasizing the importance of psychological questionnaires in the field of skills research [47].

The scientific literature highlights how the development of positive behavioral skills becomes critical during adolescence [46], with the ages 15–18 being a crucial window for the development of social and psychological skills, with this period characterized by the most intensive processes of personal development [6]. It has been found that the development of positive behavioral skills enables individual self-efficacy to increase, interpersonal skills to become stronger, problematic and aggressive behavior to decrease, and, consequently, relationships with peers and adults to improve [48,49]. The development of prosocial behavior skills plays an essential role during this period, with one study suggesting that prosocial behavior represents a key element in the development of reciprocal social relations [50]. This means that the development of positive behavioral skills is recognized as an essential factor that enables teenagers to become productive members of society [10].

The process of developing positive behavioral skills is focused on the interaction between the individual and a specific environment (e.g., team, family, school), enabling this experience to be used to integrate into other environments [51]. It has been claimed that behaviors developed during childhood do not guarantee the same behavior during adolescence [6], which means that developing various skills remains an important factor during adolescence, even if those skills were already developed during childhood. However, for positive behavior skill development programs for adolescents to be successful, it is necessary to determine at which stage of adolescence the expression of positive behavioral skills is lowest. Notably, most of the research on this topic has been conducted on the whole of the adolescence stage, with no focus on specific periods of adolescence, an issue that this study seeks to address.

This study’s relevance is further enhanced by the fact that it was conducted during the COVID-19 pandemic, thus revealing the possible impact of the pandemic on the expression of the skills under investigation. Due to COVID-19 restrictions, including the closures of schools, sport schools, parks, recreation centers, and the cancellation of youth sports, the “youth was at an increased risk for sedentary behaviors that influence their health, well-being, and academic performance.” [52] (p. 437), and a variety of social problems emerged. Apart from in-person sport activities, youths have fewer opportunities to build relationships and gain additional social support [52]. Changes in youth activities—especially in relation to activities with peers and other adults—have led to varying degrees of social isolation [53], and the social isolation experienced by many children had a significant impact on children’s positive behavioral skills [54]. Pandya and Lodha [55] found that excessive screen time during COVID-19 (during lockdown periods) was negatively associated with a variety of social problems and decreased levels of positive behavioral skills. The phenomenon of gadget addiction was a side effect of the COVID-19 pandemic due to a large-scale social restriction policy [56]. “With this policy, the intensity of playing gadgets increases so that there are gadget addictions that have implications for changes in students’ social behavior.” [56] (p. 1). Previous research by Oliveira Major, Palos, and Silva [57] found that youths who attended after-school programs showed higher levels of self-control and assertion skills, and after-school programs attendance variables had distinct impacts on social skills and behavioral problems. Findings by Bates, Greene, and O’Quinn [52] revealed that the virtual sport-based positive youth development activities facilitated positive emotional responses, positive peer interaction, engagement with family, and the utilization of environmental resources during the COVID-19 pandemic and suggested that virtual sport-based positive youth development activities “may similarly facilitate life skill transfer; an important developmental mechanism for learning in lieu of the decreased opportunities for sport and social interaction during the COVID-19 pandemic” [52] (p. 438).

Meanwhile, although previous studies have also focused on comparing the expression of skills between male and female athletes, extant research data document a greater expression of aggressive and risky behavior by male adolescents, indicating that comparative studies (by gender) can produce inaccurate conclusions, and sports-based skills development programs may not be applied at the most appropriate period of adolescence for athletes of different genders. This study’s relevance is further strengthened by the analysis of the set of skills that have been identified, such as the structural positive behavioral skills model proposed by the authors of the present study [44].

This study aims to fill several gaps in the existing research. First, positive behavioral skills have only been studied among non-sporting adolescents during the pandemic. Because positive behavioral skills are gender-specific, only a sample of boys who play sports was selected [58].

Second, adolescents who play sports were not analyzed, and no comparison was made between U16 (age 15–16 years) and U18 (age 17–18 years) athletes. The understanding that this age period (age period from 15 to 18 years) “is a developmental period marked by profound physical and psychological change, and there is a growing body of literature that recognizes adolescence as a critical period for the development of prosocial behavior skills and empathy” [59] (p. 2) enabled the establishment of the study’s main objective.

Third, the present study fills the gap in the literature by investigating the most important predictors (empathy and the demographic factor of age) of prosocial behavior with teammates and with opponents, adopting these observations as key indicators of positive behavioral skills for U16 and U18 male basketball players in the pandemic context.

The main aim of this study was to reveal the peculiarities of the expression of positive behavioral skills among U16 and U18 male basketball players during the COVID-19 pandemic. Based on the analysis of the scientific literature, it was hypothesized that the U18 male basketball players would demonstrate a greater expression of positive behavioral skills than the U16 athletes.

The secondary aim of the present study was to reveal the most important predictors (empathy and the demographic factor of age) of prosocial behavior with teammates and with opponents, adopting these observations as the most important indicators of positive behavioral skills for U16 and U18 male basketball players. This secondary aim of the study was justified on the basis of the following findings concerning adolescents in the age range 13–18 years: “The COVID-19 pandemic has had detrimental effects on adolescents’ empathy and prosocial attributes, and special attention should be given to these two longitudinally associated factors in any social crisis, such as the COVID-19 pandemic, considering their importance for adolescents’ physical, mental, and social development”. [59] (p. 1).

The secondary hypothesis was based on the results of a previous study [58] that revealed that empathy was consistently related to subsequent prosocial behavior and “for boys, levels of prosocial behavior were stable until age 14, followed by an increase until age 17” [58] (p. 2), suggesting that age and empathy possibly predict prosocial behavior with teammates and with opponents among U16 and U18 male basketball players, including during the COVID-19 pandemic.

## 2. Materials and Methods

### 2.1. Study Design

A cross-sectional survey design was chosen for the study.

### 2.2. Study Participants, Procedure, and Measures

According to the data from the list of Lithuanian basketball sports schools that were available at the time of organizing the research, there were 1401 cadets and 1546 juniors playing sports at basketball schools, making a total of 2947 young athletes. When the size of the population is known, the formula proposed by Schwarze [60] can be used to determine the sample size:

$$n=\frac{1.96^{2}Npq}{\varepsilon^{2}\left(N-1\right)+1.96^{2}pq}$$

where N is the population size. As it is known that there are 2947 young players in basketball schools, N = 2947. The value of 1.96 corresponds to the 95% confidence level of the standardized normal distribution. *p* is the expected probability of the outcome of the event that the trait in question will occur in the population under study; usually the worst-case probability of the trait occurring in half of the population is chosen (50%), and *p* = 0.5. *q* is the probability of the trait in question not occurring in the population under study (*q* = 1 − *p* = 0.5). ε is the desired precision, usually ε = 0.05. Calculating the sample size of the study according to this formula results in a minimum of 339 young athletes (n = 339) tested in the present study.

The following young athletes’ age categories were established according to the Lithuanian Basketball Federation: U16 (Under-16, cadets—athletes with a passport age of 15–16 years) and U18 (Under-18, juniors—athletes with a passport age of 17–18 years). These groups differ in the ratio of sport activities (training sessions and competitions). The ratio for training sessions per week was 3 sessions for the U16 players, and 4 sessions for U18 players, and plus the competition match (not every week due to the pandemic and possible cases of COVID-19 among players) in both cases. It should be noted that U16 and U18 adolescent athletes were allowed to have their sports activities in face-to-face contact only from the end of January 2021 in Lithuania during the pandemic after lockdown.

A two-stage cluster sampling procedure was used, whereby the required number of basketball schools was first selected by lottery from a list of sports schools (the first stage of the selection process). As each basketball school has at least 45 players among U16 (age 15–16 years) and U18 (age 17–18 years) age categories, it was planned to select 11 sports schools and to invite more than 400 players. However, two sports schools did not agree to take part in the study. Subsequently, all male U16 and U18 players at the selected basketball schools (second stage of selection) participated in a survey.

This study included young basketball players from nine Lithuanian basketball schools (400 players were invited and 8 declined participation). After conducting surveys, which were conducted in selected teams from selected basketball schools before their training sessions, 392 completed questionnaires were received. Data from 14 participants were excluded due to unclear marking or some answers not being marked at all. This meant that the data for 378 young basketball players were included in the analysis. The study was conducted during the period from December 2021 to January 2022. During this period, the COVID-19 pandemic persisted, meaning various pandemic restrictions on social contact remained in place. The anonymity and confidentiality of the research data were ensured during the study. No information was required by the questionnaires that could identify the subjects. Prior to starting the study, permission was obtained from the Ethics Committee for Social Research of the Lithuanian Sports University (Approval No. SMTEK-47, Approval Date: 3 June 2021). Prior to conducting the survey for the study, permission was obtained from the sports school administrations to perform the research.

During the study, young basketball players completed the self-administered survey, which included sociodemographic measures (i.e., age) and validated instruments for usage in Lithuania to measure positive behavioral skills (the instruments detailed above). The opening section of the survey included general information on the study and a statement with regard to consent to participate in the study.

### 2.3. Sociodemographic Variables

The study sample comprised 378 young male basketball players, with an average age of 16.36 ± 1.15 years. The study included 46.8% (N = 177) of cadets (U16—athletes aged 15–16) and 53.2% (N = 201) of junior (U18—athletes aged 17–18) basketball players.

### 2.4. Structural Model of Positive Behavioral Skills

The analysis of the scientific literature regarding positive behavioral skills among young athletes in the previous study [44] allowed the following groups (categories) of positive behavioral skills to be identified: positive personal, positive social, and positive emotional skills. Furthermore, a set of positive behavioral skills has been assigned to each of these categories. The positive personal skills category includes the following skills: taking personal responsibility, self-esteem, prosocial behavior with teammates, and prosocial behavior with opponents [44]. Positive social skills include taking social responsibility, cooperation, assertiveness, empathy, and self-control [44]. Positive emotional skills include the ability to evaluate and convey emotions, the ability to utilize one’s positive emotional experience, the ability to comprehend and analyze emotions, and the ability to control emotions [44]. This classification scheme is called the structural model of positive behavioral skills [44].

### 2.5. Instruments for Measuring Positive Personal Skills and Social Responsibility Skills

#### 2.5.1. Personal and Social Responsibility Questionnaire

To assess personal and social responsibility skills, the Personal and Social Responsibility Questionnaire (PSRQ) [61] was selected. This questionnaire comprises 14 statements and features two scales: a personal responsibility scale (seven statements) and a social responsibility scale (seven statements). The personal responsibility scale was used to assess positive personal skills, and the social responsibility scale was used to assess positive social skills. All questionnaire statements were rated on a six-point Likert scale ranging from 1 (strongly disagree) to 6 (strongly agree) [61,62]. The reliability and construct validity of the PSRQ are well established [61]. Evidence of external validity of the Lithuanian version of the PSRQ are the results of the study with two groups among athletes of 16.87 years old (standard deviation *SD* = 0.24) and 16.96 years old (*SD* = 0.32) when “convergent validity was accepted for both constructs, given that the average variance extracted (AVE was used to evaluate convergent validity) values of each construct were 0.52 and 0.54, respectively” [62] (p. 324). Factorial structure of PSRQ was stable within the two independent samples, and “this is interpreted as being an indication of cross validity” [62] (p. 325).

The internal consistency of the Lithuanian version of the PSRQ was tested with the U16 and U18 age groups [63], and the subscales demonstrated acceptable levels of internal consistency, with Cronbach’s *α* ranging from 0.68 for personal responsibility to 0.75 for social responsibility. Construct validity was assessed “through confirmatory factor analysis, which showed that the expected factor structure is correct: χ2 (76) = 147.93, *p* < 0.0001; NNFI = 0.92; CFI = 0.93; RMSEA = 0.06” [63] (p. 291). Meanwhile, the Cronbach’s *α* of these scales for the current study sample were 0.85 for personal responsibility and 0.78 for social responsibility.

#### 2.5.2. Rosenberg Self-Esteem Scale

The Rosenberg self-esteem scale (RSES) was chosen to assess positive self-esteem expression. The RSES questionnaire comprises ten statements that are rated on a four-point Likert scale ranging from 0 (strongly disagree) to 3 (strongly agree). The final scores are interpreted as follows: scores of 0–10 indicate low self-esteem, scores of 11–20 indicate moderate self-esteem, and scores of 21–30 indicate high self-esteem [64,65].

There is extensive evidence of the reliability and validity of the scale [65]. “Test–retest reliability over a period of 2 weeks reveals correlations of 0.85 and 0.88, indicating excellent stability” [64] (p. 61). The questionnaire was translated into Lithuanian and was used in the Health Behavior in School-Aged Children study, coordinated by the WHO; Cronbach’s alpha = 0.75 [66].

According to a previous study, “The Lithuanian version of the RSES has a reported internal consistency of 0.73.” [67] (p. 64). In the study with the male athletes of U16 and U18 age groups, a Cronbach’s alpha of 0.72 was found for the RSES total score, indicating acceptable internal reliability [67]. Evidence of external validity of the Lithuanian version are the results of the study with the cohort of students in the final year of secondary school and one year later during the first year of university (mean age at the start of the study was 18.54 years (*SD* = 0.78)). Correlations between psychological wellbeing scale scores (RPWBS) and RSES scores have been established, with positive statistically significant correlation coefficients: r = 0.14 (at the start of the study) and r = 0.17 (one year later) [68]. The internal consistency value (Cronbach’s *α*) of the RSES for the current study sample was 0.61.

#### 2.5.3. The Prosocial and Antisocial Behavior in Sport Scale

The prosocial and antisocial behavior in sport scale (PABSS) was used to assess prosocial behavior skills in sports [18]. The questionnaire comprises four scales, but only two were used in this study: the prosocial behavior with teammates scale (four items) and the prosocial behavior with opponents scale (three items). This approach was adopted because the purpose of this research was the analysis of positive behavioral skills. Each questionnaire item is rated on a five-point Likert scale ranging from 1 (never) to 5 (very often) [18,69]. Evidence of previous study [69] “supported the convergent, concurrent, and discriminant validity of the scale, provided evidence for its test–retest reliability and stability, and suggest that the instrument is a valid and reliable measure of prosocial and antisocial behavior in sport” [69] (p. 1208). The Lithuanian version of the PABSS had been adapted and validated with student athletes [70]. Factor analysis of the PABSS revealed a four-factor solution similar to those of the original scale version. “Distinguishing of the four factors (scales) similar to those of the original scale version was interpreted as an indication of the instrument’s construct validity” [70] (p. 99). Concurrent validity was tested using the Youth Values in Sport Questionnaire. It was revealed that “moral values positively related to prosocial behavior toward teammates (r = 0.34) and opponents (r = 0.30) and negatively related to antisocial behavior toward opponents (r = –0.29)” [70] (p. 104). The Lithuanian version of the PABSS has demonstrated adequate internal consistency, with Cronbach’s α between 0.79 and 0.85 [70]. The internal consistency of the Lithuanian version of the questionnaire has been tested with the U16 and U18 age groups, producing Cronbach’s α ranging from 0.79 to 0.84 [71]. For the present sample, Cronbach’s α for prosocial behavior with teammates was 0.86, and Cronbach’s α for prosocial behavior with opponents was 0.80.

### 2.6. Instrument to Assess Positive Emotional Skills

The Schutte Self-Report Inventory (SSRI) was selected to assess positive emotional skills. The questionnaire comprises 33 statements and features four scales: the ability to assess and express emotions (8 statements), the ability to use one’s positive emotional experiences (14 statements), the ability to understand and analyze emotions (6 statements), and the ability to control emotions (5 statements). Each statement on the questionnaire is rated on a five-point Likert scale ranging from 1 (strongly disagree) to 5 (strongly agree) [72,73]. The Lithuanian version of the SSRI has shown an internal consistency of 0.79 and a test–retest reliability coefficient of 0.84 for the overall questionnaire, following testing with U16 and U18 age groups [74]. External validity of the Lithuanian version of the SSRI has been tested using “comparisons of the overall questionnaire scores, which confirm the absence of significant mean difference and small effect size (Cohen’s *d* = 0.09) between the English and Lithuanian versions of the SSRI” [75] (p. 5). Concurrent validity of the Lithuanian version of the SSRI was tested in the sample of basketball players using the sport motivation scale (SMS-II), and positive significant correlations were found between all scales of the SSRI and “intrinsic, integrated, and identified regulation. Amotivated regulation was significantly negatively correlated (r = –0.19) with total SSRI score” [76] (p. 6).

The following internal consistency reliability estimates (Cronbach’s α) were observed for the present sample: the ability to assess and express emotions: 0.68; the ability to use one’s positive emotional experiences: 0.74; the ability to understand and analyze emotions: 0.77; the ability to control emotions: 0.66.

### 2.7. Instrument for Measuring Positive Social Skills

The Social Skills Rating System-Secondary Student form (SSRS-S) [77] was chosen to assess positive social skills. It comprises thirty-nine statements, with a certain number assigned to each of the four scales: cooperation scale (ten statements), assertiveness scale (nine statements), empathy scale (ten statements), and self-control scale (ten statements). Each statement is rated on a three-point Likert scale from 0 (never) to 2 (very often) [77,78].

In the sample of 172 students (aged 15 years (*SD* = 0.72)) the test–retest reliability for the overall scale was 0.81 [79]. The internal consistency of the Lithuanian version of the questionnaire was tested with the U16 and U18 age groups [80]. Cronbach’s α for the Lithuanian version of the SSRS-S has been observed to range from 0.66 to 0.76. [80]. The internal consistency of the Lithuanian version of the PSRQ was also tested with the 15–16 year old adolescent athletes, and the subscales demonstrated acceptable levels of internal consistency, with Cronbach’s α ranging from 0.67 to 0.68 [81]. The external validity of the adapted Lithuanian version of the questionnaire was checked in the framework of the project in a national population of 5–10 grade students (N = 2916) by Griciūtė et al. [82], and was found that the adapted Lithuanian version of SSRS-S has a strong external validity of the subscales. For the current study sample, the internal consistency values (Cronbach’s α) for this questionnaire’s scales were as follows: cooperation: 0.75; assertiveness: 0.68; empathy: 0.77; self-control: 0.73.

### 2.8. Statistical Analysis

The research data were analyzed using IBM SPSS Statistics version 28.0, a statistical software package for social sciences. The normality of the variables was checked (using calculations of skewness and kurtosis values, which were all between −1 and 1), and then means, standard deviations, mean differences (*D*s), and Pearson’s correlations were calculated for the study variables. The independent samples *t*-test was used to determine the equality of means between groups. Differences in values were considered statistically significant if the probability value of the error was *p* < 0.05, with 95% reliability, and *p* < 0.01, with 99% reliability. A set of two hierarchical (stepwise) regression analyses were used to reveal the most important predictors (empathy and the demographic factor of age) of prosocial behavior with teammates and prosocial behavior with opponents, adopting these as key indicators of positive behavioral skills for U16 and U18 male basketball players. The first regression step included empathy, and the second step included empathy and age. The internal consistency of the questionnaire scales used in the study was calculated using Cronbach’s α coefficient. The effect size was calculated using Cohen’s *d* criterion [83]. Cohen’s *d* effect sizes are generally defined as small (*d* = 0.2), medium (*d* = 0.5), and large (*d* = 0.8). Cohen’s *d* can be considered a kind of standardized score.

## 3. Results

Calculation of Pearson’s correlations between study variables, namely between positive behavioral skills and participants’ age (Table 1), revealed the highest statistically significant correlation between participants’ age and self-esteem (0.511), indicating that self-esteem increases with age. Positive and statistically significant correlations were also observed between age and taking personal responsibility (0.370), taking social responsibility (0.299), the ability to control emotions (0.262), cooperation (0.294), assertiveness (0.150), self-control (0.136), and prosocial behavior with teammates (0.118). Positive but statistically insignificant correlations were observed between age and the ability to utilize one’s positive emotional experience (0.063), the ability to comprehend and analyze emotions (0.067), empathy (0.063), and prosocial behavior with opponents (0.043). A statistically insignificant correlation was observed between age and the ability to evaluate and convey emotions (0.027).

The independent samples *t*-test and Cohen’s *d* effect size calculation enabled the evaluation of the expression of positive behavioral skills in the different age categories of basketball players: U16 (age 15–16 years) and U18 (age 17–18 years) (Table 2). The data analysis revealed that U18 basketball players exhibit higher scores for the expression of positive behavioral skills than U16 basketball players. Cohen’s *d* effect sizes ranged from weak (−0.13) to medium (−0.79) across all scales. Statistically significant results were observed between the two groups for taking personal responsibility (*D* = 3.2; *p* < 0.001), taking social responsibility (*D* = 2.9; *p* < 0.001), the ability to control emotions (*D* = 1.92; *p* < 0.001), cooperation (*D* = 1.82; *p* < 0.001), assertiveness (*D* = 0.85; *p* < 0.001), self-control (*D* = 0.77; *p* = 0.02), self-esteem (*D* = 1.89; *p* < 0.001) and prosocial behavior with teammates (*D* = 0.18; *p* = 0.01), and U18 (age 17–18 years) basketball players exhibit higher scores than U16 (age 15–16 years) athletes. This means that U18 athletes are more capable of taking personal responsibility and positively evaluating themselves, and are more inclined to behave pro-socially toward their teammates and take social responsibility. They also exhibit higher levels of assertiveness, self-control, and emotional control than U16 athletes.

As mentioned, compared to players from the U16 group, participants from the U18 group also demonstrated higher but statistically non-significant results for the indicators of ability to evaluate and convey emotions, ability to utilize one’s positive emotional experience, ability to comprehend and analyze emotions, empathy, and prosocial behavior with opponents.

For the first regression analysis––with prosocial behavior with teammates as the dependent variable––introducing empathy as the predictor at Step 1 revealed a significant predictive effect, *F* (1, 376) = 89.77, *p* < 0.001; R^2^ = 0.19 (Table 3).

At Step 2, the addition of age produced a significant increase in variance explained (R^2^-change = 0.01, *F* (1, 375) = 4.97, *p* = 0.026), suggesting that age contributed a significant amount of predictive value for the dependent variable. The overall model of the simple regression analysis (adjusted R^2^ = 0.20) explained 20% of the variance in basketball players’ empathy scores in the study context (an effect-size index considered small).

For the second regression analysis with prosocial behavior toward opponents as the dependent variable (Table 3), the addition of empathy as a predictor at Step 1 yielded a significant effect (R^2^ = 0.16, *F* (1, 376) = 73.98, *p* < 0.001). At Step 2, the addition of age did not indicate a significant increase in variance explained (R^2^-change = 0.001, *F* (1375) = 0.45, *p* = 0.505), suggesting that age did not contribute a significant amount of predictive value for the dependent variable (prosocial behavior with opponents).

## 4. Discussion

This study’s main aim was to reveal the characteristics of the expression of positive behavioral skills in U16 and U18 male basketball players. The methods of data analysis with a representative sample were applied to reveal that U18 adolescent basketball players have more developed positive behavioral skills, confirming the study’s first hypothesis. In addition, this study assessed the set of skills that comprise the positive personal skills construct, which is called the structural model of positive behavioral skills. The study revealed statistically significant differences in skill scores between the groups of participants in the areas of taking personal responsibility, self-esteem, and prosocial behavior with teammates, with these being higher for the U18 age group. The data analysis enabled the conclusion that the U18 participants are more adept at taking personal responsibility than the U16 adolescent basketball players.

The present study revealed that U18 basketball players demonstrate statistically significantly higher levels of prosocial behavior with teammates (effect size is small; *d* = −0.26). Although not statistically significant, U18 players also scored higher for prosocial behavior with opponents. These results are consistent with previous studies. For example, an analysis of prosocial behavior with teammates among U16 and U18 soccer players showed that U18 players demonstrate a more developed prosocial behavior with teammates (effect size is small; *d* = −0.27), whereby their prosocial behavior toward opponents do not differ significantly [71].

This study also assessed the expression of skills comprising the positive emotional skills construct in basketball players from different age categories. Statistically significant results for the ability to control emotions were only found between the two age categories, with U18 participants recording higher scores for this skill (effect size is medium; *d* = −0.65). This is supported not only by the results of this study, but also by the data of other authors [84], which reveal that younger adolescents have less ability to control their emotions (effect size is medium; *d* = 0.49).

The results of the present study align with those of other studies [85] that found that basketball players in the older age categories demonstrate a greater emotional stability and stronger self-regulation. Another study [86] on emotional control skills comparing U16 and U18 players documented no statistically significant differences, with the U18 age group scoring marginally higher.

This study also assessed the positive social skills of young basketball players. The findings revealed that adolescent basketball players in the U18 age category produced statistically significantly higher scores for taking social responsibility, cooperation, assertiveness, and self-control compared to players in the U16 age category (effect size ranges from small to medium). In the current study, statistically significant differences were observed between the social responsibility skill levels of the U16 and U18 players, with higher scores among the U18 age group (effect size is medium; *d* = 0.63). The results of the present study align with those of [71], whose authors investigated the social responsibility skills of U16 and U18 football players. Those authors found that U18 footballers demonstrate higher levels of social responsibility skills (effect size small; *d* = 0.27) [71].

According to the current study, U18 basketball players demonstrate stronger cooperation skills than U16 basketball players (effect size is medium; *d* = −0.63.), aligning with the findings of a study [21] that produced results suggesting that basketball is a sport that promotes cooperation. Based on the results of the present study, U18 basketball players show higher levels of assertiveness than U16 basketball players, with a small effect size (*d* = −0.30). These findings are consistent with those of a study on assertiveness among U16 and U18 football players, which found that U18 football players exhibited higher assertiveness scores than U16 football players (effect size is small; *d* = −0.10) [71].

Furthermore, in this study, adolescent basketball players in the U18 age group recorded statistically significantly higher self-control skill scores (effect size is small) than participants in the U16 age group. Researchers [87] who studied the self-control skills of adolescents from different age groups (U16 and U18) found that adolescents in the U18 age group who participated in sports demonstrated statistically significantly higher levels of self-control (effect size is small; *d* = −0.45), findings which were consistent with the results of the present study.

Notably, the development of positive behavioral skills among U16 basketball players may have been influenced by the negative experience of the COVID-19 pandemic [88,89]. During the COVID-19 period, adolescents were severely and negatively affected by measures such as the closure of schools and other institutions, which encouraged social disengagement [90] and led to a significant decline in adolescents’ educational progress [91]. A possible negative effect of the pandemic on the athletes’ positive behavioral skills under investigation was confirmed by the reduced ability of the U16 players to control their emotions, because in the current study, U16 participants recorded lower scores for the ability to control emotions than U18 players. Moreover, the effect size of this study was medium during the pandemic but small before the pandemic for the differences of these abilities according to other researchers’ findings [81]. Stronger differences were observed during the pandemic between the level of the social responsibility skills among U16 participants compared to those of U18 (effect size was medium during the pandemic in the present study and was small in other researchers’ findings [71] before the pandemic). The same pattern holds true for the cooperation skills—U16 participants recorded lower scores for the cooperation skills than U18 players and effect size was medium during the pandemic, but in other researchers’ findings [86,87] before the pandemic, the effect size was small for the differences of these skills.

The second hypothesis––that age and empathy predict prosocial behavior––has been partially confirmed. Empathy was a strong predictor of prosocial behavior with regard to opponents, but unexpectedly, age did not predict prosocial behavior with opponents. This supports the findings of Bruner et al. [92] that “this may have been accounted for by infrequent opportunity for such acts” [92] (p. 61) during the pandemic (due to quarantine limiting the ability to compete). It should also be noted that there were no significant differences in age in the current sample (16.36 ± 1.15) or in the study sample considered by Bruner et al. [92] (M = 15.88). However, further empirical research is needed to explain this possibility and to confirm or refute it.

During the COVID-19 pandemic, the sports sector was among the most affected and restricted, and athletes involved in team sports had to wait the longest for the lifting of the pandemic restrictions, meaning that team sports were the most affected by those restrictions [93].

### Strengths and Limitations

One strength of this study is that a wide range of positive behavioral skills were analyzed. Another strength is that it was conducted during the COVID-19 pandemic, enabling the identification of patterns of positive behavioral skills among U16 and U18 basketball players during this period, thereby contributing to filling the gaps in the scientific evidence concerning the possible effects of COVID-19 on adolescent athletes.

One weakness of this study is that only male adolescent athletes were examined. The sample did not include both males and females because various different scientific studies have confirmed the differential expression of positive behavioral skills between boys and girls. Combining data from males and females can produce false research conclusions.

Another limitation is that this cross-sectional study only used survey methods. Future research should include an educational experiment. Furthermore, future studies should focus on younger adolescent athletes because the current study has observed U16 athletes scoring lower for all skill indicators, confirming the study hypothesis. It is possible that younger adolescent athletes will demonstrate even lower scores for indicators of positive behavioral skills. Based on this study’s results, future researchers can develop positive behavioral skills development programs for sports and adapt them to specific periods of adolescence.

## 5. Conclusions

This study fills a gap in the existing research by providing insight into the peculiarities of the wide set of positive behavioral skills demonstrated by U16 and U18 male basketball players during the COVID-19 pandemic.

It has been revealed that U18 adolescent athletes are more capable of taking personal responsibility, positively evaluating themselves, behaving pro-socially with teammates, taking social responsibility, cooperating, demonstrating assertiveness, demonstrating self-control, and managing emotions than U16 adolescent athletes. When comparing the effect sizes in our study during the pandemic with similar studies by other authors, the pandemic may have had a larger negative effect on some positive behavioral skills (ability to control emotions, social responsibility skills, cooperation skills) in U16 athletes than in U18 athletes, as the effect sizes were small before the pandemic and moderate during the pandemic (in the current study).

Empathy, in conjunction with age, were powerful predictors of prosocial behavior with teammates. Age contributed a significant amount of predictive value for this dependent variable. Meanwhile, although empathy strongly predicted prosocial behavior with opponents, the age of players did not contribute a significant amount of predictive value for prosocial behavior with opponents.

This study’s results should encourage researchers from different parts of the world to investigate positive behavioral skills in the context of adolescent athletes. Given that the COVID-19 pandemic has affected athletes worldwide, and given the importance of sports in terms of skill development, it is imperative to investigate the level of positive behavioral skills of adolescent athletes in the post-pandemic context.

This study’s conclusions could be useful for developing positive behavioral skills training programs for young athletes that focus on the skills examined in this study. The development of positive behavioral skills training programs should focus primarily on athletes in the U16 age category because athletes from this age group have been observed to record lower levels of positive behavioral skills. In addition, the added value of this study is that its results (obtained during the pandemic) will allow future researchers to make comparisons to reveal trends in the levels of positive behavioral skills in the post-pandemic period.

## Figures and Tables

**Table 1:** Correlations of study variables.

	1	2	3	4	5	6	7	8	9	10	11	12	13	14
1. Taking personal responsibility	1													
2. Taking social responsibility	0.702 **	1												
3. Ability to evaluate and convey emotions	0.008	0.087	1											
4. Ability to utilize one’s positive emotional experience	0.091	0.091	0.470 **	1										
5. Ability to comprehend and analyze emotions	0.062	0.068	0.448 **	0.683 **	1									
6. Ability to control emotions	0.135 **	0.108 *	0.410 **	0.614 **	0.579 **	1								
7. Cooperation	0.109 *	0.105 *	0.240 **	0.364 **	0.310 **	0.385 **	1							
8. Assertiveness	−0.008	0.016	0.264 **	0.424 **	0.420 **	0.480 **	0.588 **	1						
9. Empathy	0.134 **	0.091	0.301 **	0.366 **	0.369 **	0.363 **	0.462 **	0.595 **	1					
10. Self-control	0.047	0.009	0.190 **	0.291 **	0.207 **	0.221 **	0.650 **	0.539 **	0.405 **	1				
11. Self-esteem	0.264 **	0.205 **	0.055	0.104 *	0.146 **	0.325 **	0.209 **	0.197 **	0.164 **	0.021	1			
12. Prosocial behavior with teammates	0.145 **	0.164 **	0.451 **	0.438 **	0.443 **	0.386 **	0.390 **	0.379 **	0.439 **	0.248 **	0.165 **	1		
13. Prosocial behavior with opponents	0.055	0.071	0.159 **	0.325 **	0.388 **	0.379 **	0.259 **	0.387 **	0.405 **	0.156 **	0.207 **	0.418 **	1	
14. Age	0.370 **	0.299 **	0.027	0.063	0.067	0.262 **	0.294 **	0.150 **	0.063	0.136 **	0.511 **	0.118 *	0.043	1

Notes. * p < 0.05; ** *p* < 0.001.

**Table 2:** Statistics for the positive behavioral skills of U16 and U18 players.

	U16 (N = 177)	U18 (N = 201)	*t* Value	*p* Value	Cohen’s *d*
Taking personal responsibility	30.01 ± 3.87	33.21 ± 4.16	−7.72	<0.001 **	−0.79
Taking social responsibility	31.28 ± 4.37	34.18 ± 4.84	−6.79	<0.001 **	−0.63
Ability to evaluate and convey emotions	28.15 ± 4.22	28.27 ± 3.58	−0.30	0.76	−0.03
Ability to utilize one’s positive emotional experience	54.74 ± 6.27	55.51 ± 5.24	−1.33	0.18	−0.14
Ability to comprehend and analyze emotions	21.76 ± 3.99	22.40 ± 2.80	−1.82	0.07	−0.19
Ability to control emotions	15.81 ± 3.03	17.73 ± 2.89	−6.32	<0.001 **	−0.65
Cooperation	13.85 ± 2.77	15.67 ± 3.01	−6.08	<0.001 **	−0.63
Assertiveness	11.67 ± 2.78	12.52 ± 2.92	−2.87	<0.001 **	−0.30
Empathy	13.83 ± 3.16	14.40 ± 3.63	−1.63	0.11	−0.17
Self-control	13.68 ± 3.08	14.45 ± 3.16	−2.39	0.02 *	−0.25
Self-esteem	15.40 ± 1.73	17.29 ± 1.48	−11.45	<0.001 **	−0.17
Prosocial behavior with teammates	4.00 ± 0.72	4.18 ± 0.63	−2.61	0.01 *	−0.26
Prosocial behavior with opponents	3.33 ± 0.90	3.45 ± 0.97	−1.24	0.22	−0.13

Notes. * *p* < 0.05; ** *p* < 0.01. U16—cadets, athletes aged 15–16. U18—juniors, athletes aged 17–18.

**Table 3:** Hierarchical regression results for prosocial behavior with teammates and with opponents.

Step	Dependent Variable	Predictor Variable (s) Entered	R^2^	R^2^-Change	*F*-Change	df1 df2	Beta
1	Prosocial behavior with teammates	Empathy	0.19	0.19	890.77 **	1 376	0.439 **
2		Empathy	0.20	0.01	40.97 *	1 375	0.430 **
		Age					0.103 *
1	Prosocial behavior with opponents	Empathy	0.16	0.16	730.98 **	1 376	0.405 **
2		Empathy	0.17	0.001	0.45	1 375	0.403 **
		Age					0.032

Notes. * *p <* 0.05; ** *p <* 0.01.

## Data Availability

The datasets collected and analyzed during the current study are available from the corresponding author upon reasonable request.
